# A new method of preoperative assessment of correct electrode array alignment based on post-operative measurements in a cochlear implanted cohort

**DOI:** 10.1007/s00405-022-07421-8

**Published:** 2022-06-21

**Authors:** Bence Horvath, Adam Perenyi, Fiona Anna Molnar, Roland Nagy, Miklos Csanady, Jozsef Geza Kiss, Laszlo Rovo

**Affiliations:** 1 Doctoral School of Clinical Medicine, University of Szeged, Szeged, Hungary; 2 Department of Oto-Rhino- Laryngology and Head- Neck Surgery, University of Szeged, Szeged, Hungary; 3 Faculty of Medicine, University of Szeged, Szeged, Hungary

**Keywords:** Cochlear implant, Slim Modiolar, Medical image processing, 3D Slicer, Neuro-otology surgery

## Abstract

**Purpose:**

During cochlear implantation surgery, a range of complications may occur such as tip fold-over. We recently developed a method to estimate the insertion orientation of the electrode array. The aim of the study was to determine the optimal angle of orientation in a cohort of cochlear implanted patients.

**Methods:**

On eighty-five CT scans (80 uncomplicated insertions and 5 cases with tip fold-over), location of the electrode array’s Insertion Guide (IG), Orientation marker (OM) and two easily identifiable landmarks (the round window (RW) and the incus short process (ISP)) were manually marked. The angle enclosed by ISP-RW line and the Cochlear™ Slim Modiolar electrode array’s OM line determined the electrode array insertion angle.

**Results:**

The average insertion angle was 45.0–47.2° ± 10.4–12° SD and was validated with 98% confidence interval. Based on the measurements obtained, patients’ sex and age had no impact on the size of this angle. Although the angles of the tip fold-over cases (44.9°, 46.9°, 34.2°, 54.3°, 55.9°) fell within this average range, the further it diverted from the average it increased the likelihood for tip fold-over.

**Conclusion:**

Electrode array insertion in the individually calculated angle relative to the visible incus short process provides a useful guide for the surgeon when aiming for the optimal angle, and potentially enhances good surgical outcomes. Our results show that factors other than the orientation angle may additionally contribute to failures in implantation when the Slim Modiolar electrode is used.

## Introduction

Cochlear implantation is a modern and effective hearing rehabilitation technique for patients with severe to profound sensorineural hearing loss [1]. The speech processor, which is worn behind the ear, detects sound and converts it into an electrical signal. The internal unit is implanted surgically to position the stimulating electrodes close to the spiral ganglion cells in the cochlea and directly stimulate them with these electrical signals. The most common procedure to advance the electrode array into the scala tympani of the cochlea is performed by the posterior tympanotomy, by opening the facial recess, via the round window (RW). The bony overhang that restricts the access to the RW membrane is usually removed. The RW anatomy is variable among individuals which in some instances requires its widening (“extended RW approach”) [2]. Possible complications of electrode array insertion are interscalar dislocation and tip fold-over [3]. Potential further hazards are formation of short circuits and implant dysfunction. Using the recommended cochlear implant soft surgery techniques [4], it is possible to preserve residual hearing and this would require the preservation of the internal structure of the cochlea.

**Fig. 1:**
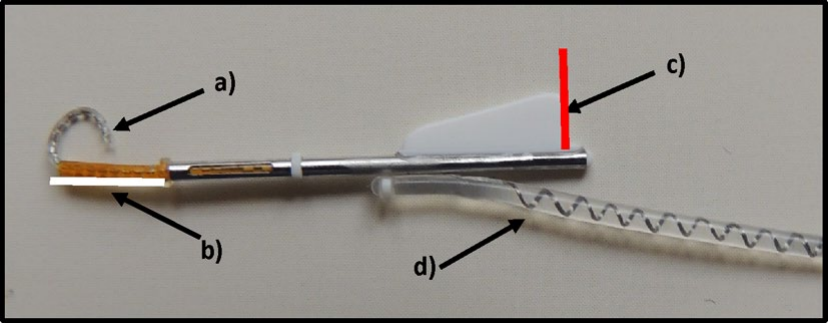
Cochlear™ Nucleus^®^ CI 532 Slim Modiolar practice cochlear implant. The major parts of the device: **a** Cochlear implant electrode, **b** Insertion Guide (IG; white line), **c** Orientation marker (OM; red line), **d** Electrode lead. These lines (white and red) are marked on the subsequent CT scan (Fig. 2a) and 3D model (Fig. 3a and b) for easier orientation

The highest proportion of the cochlear implants (CI) that were used since 2015 at the Department of Otorhinolaryngology, Head and Neck Surgery, University of Szeged, based on preferable low-trauma procedure and audiological benefits [5] have been Cochlear™ Nucleus^®^ CI532 (Fig. 1.) and CI632. Both devices are mounted with one of the thinnest perimodiolar electrode arrays (Slim Modiolar) [6]. Perimodiolar electrode arrays are pre-curved and this property supports their close-to-modiolus or ‘modiolus hugging’ position. In our clinic, the perimodiolar electrode array is preferred over the straight and the thicker Contour Advance arrays because of the lower energy consumption for stimulation and less trauma to the cochlea [7]. However, tip fold-over of the Slim Modiolar electrode has been reported with a higher incidence than for any other electrode arrays [8]. One of the possible reasons for tip fold-over is the unfavourable orientation of the electrode array adopted during insertion [8].

**Fig. 2:**
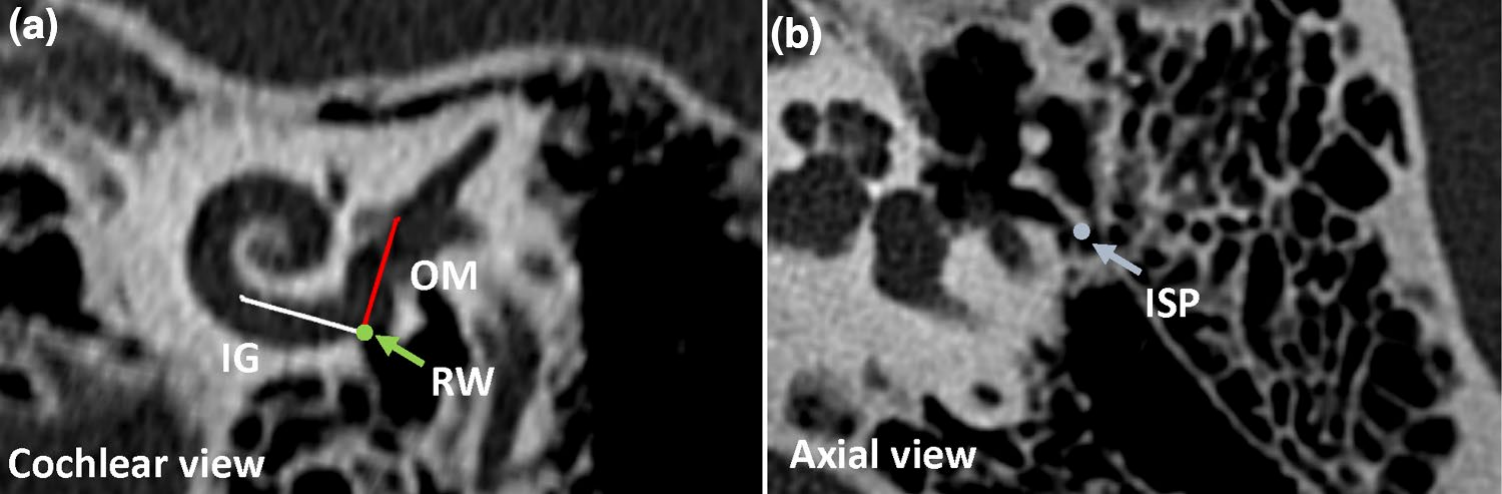
**a** The lines that represent the parts of the cochlear implants as introduced on Fig. 1: white line: insertion guide (IG), red line: orientation marker (OM). These lines were drawn and cross each other in the round window (RW; green dot). **b** The incus short process (ISP) is indicated on the axial plane (blue)

**Fig. 3:**
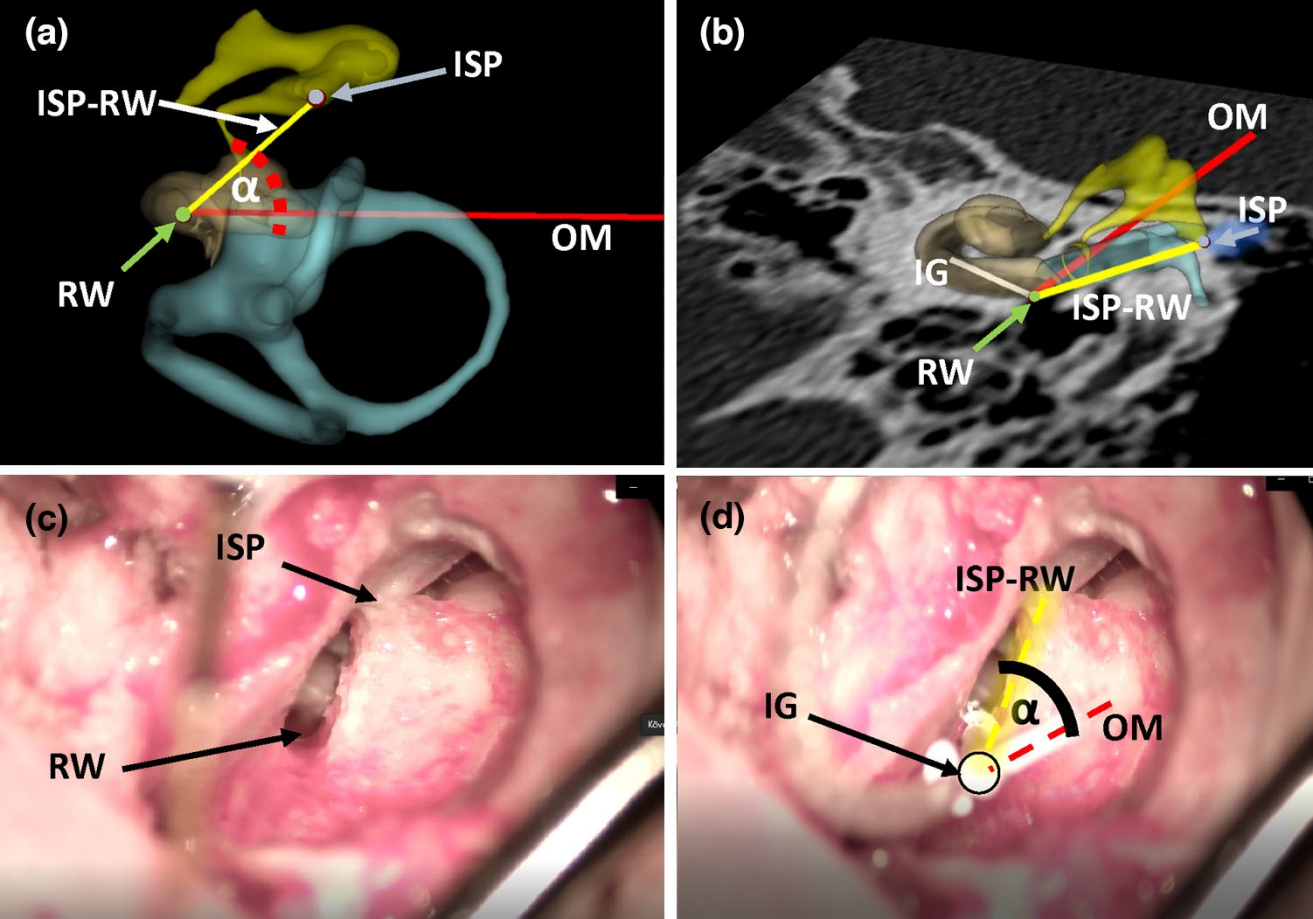
**a** A 3D model that illustrates the lines and the anatomical structures in the anterior view, α is the calculated angle, in this case *α* = 45.07°. Red line represents OM, yellow line represents the reference ISP-RW line that links the incus short process (ISP) and ends in the round window (RW). IG is perpendicular to the round window. **b** A 3D model in an inferior view, showing the location of each previously mentioned lines on one CT slice (Fig. 2; cochlear view). **c** A surgical image of the view during implantation; the ISP and RW are marked. These are chosen as measurements, because these anatomical landmarks are clearly visible during surgery. **d** The identical surgical image with the CI electrode, IG is closely perpendicular to RW. The line of OM and the line reference ISP-RW are indicated (dashed lines). These lines enclose α, the angle to be determined

The aim of the study was to determine the ideal angle of orientation by calculating the proximity of the electrode array to intraoperatively visible anatomical landmarks, visualised by CT scan, in a cohort of cochlear implanted patients.

## Materials and methods

All patients who had severe to profound hearing loss and received cochlear implantation with a Slim Modiolar device (Cochlear™) between January 2016 and September 2021 (Table 1) were included in this study. Ethical approval was obtained from the local ethical research committee, University of Szeged, Szent-Györgyi Albert Medical Center, Regional and Institutional Human Biomedical Research Ethics Committee (Szegedi Tudományegyetem, Szent Györgyi Albert Klinikai Központ Humán Orvosbiológiai Regionális és Intézmenyi Kutatásetikai Bizottság) and the appropriate informed consent was obtained from patient or guardian. Our inclusion criteria of CT scans included: good quality high-resolution CT scans of the temporal bones and clear visible structure of the cochlea and auditory ossicles (high-resolution thin slices up to 0.625 mm, with no motion artefact). Exclusion criteria were cochlear malformations [9], cochlear ossification, and obliterative post meningitis changes. Eighty consecutive CT scans of cochlear implanted patients who underwent uncomplicated implantation with Slim modiolar electrode and complied with the above criteria were analysed to determine the ideal insertion angle. Preoperative CT scans of five patients with electrode array tip fold-over were additionally analysed.

**Table 1:** Distribution of cases by sex and age

	Count	Youngest [years]	Oldest [years]	Average [years]	Standard deviation [years]
Female	36	1	77	24.1	25.4
Male	44	1	75	21.5	24.5
All	80	1	77	22.7	24.8

The required quality of the CT scans and the normal anatomy of the selected patients were confirmed by a radiologist with a subspecialisation in head and neck imaging. These images were processed by an open source free image visualization software: 3D Slicer (version: 4.10.1, operating system Win10) [10], which is available on all platforms (Win, Mac, Linux). After having imported the DICOM files, we converted the image series into single “nrrd” files, the proprietary file format of 3D Slicer. This conversion process anonymized the images, after which the images did not contain any personal information of the patients. This conversion does not introduce distortion or any anomalies into the image.

Calculations were carried out as described in our previous paper [11]. In brief, on cochlear view (Fig. 2a) [12] in which the basal turn of the cochlea is best seen, two straight lines were drawn. The first line represents the insertion guide (IG; white) of the CI and the second line is the orientation marker (OM; red) shown in Fig. 2a. These two lines are perpendicular to each other and intersect at the round window. This view is the plane of the ideal electrode array insertion.

Then the tip of the incus short process (ISP) was marked (Fig. 2b; blue dot) on the axial CT scan and then connected with a virtual line to the round window (ISP-RW, Fig. 3). This line was projected into a common plane with the line of OM. We compared the position of the OM to this ISP-RW virtual line (Fig. 3a; angle α).

The above-mentioned three parameters (lines of IG and OM, ISP) are sufficient to calculate the ideal alignment of the OM and were chosen as measurements, because they are clearly visible during surgery (Fig. 3c and d). The RW was also marked, but its coordinates were not used for the calculations. We created a custom scripted module in 3D Slicer. The 3D coordinates of OM, IG and ISP were loaded into this module to determine the angle enclosed by the line of OM and virtual ISP-RW line. The spatial location of the selected structures during surgery is illustrated in Fig. 3.

## Results

### Basic statistical analysis of the electrode array insertion angles

From eighty different patients, we determined the average angle using our previously developed method [11]. The calculations were performed using our python scripted 3D Slicer module. The results obtained by the program were confirmed by manual measurement of the first five patients’ angles (left and right sides). Since the manual calculations were equal to the values obtained by the module, we accepted that the program worked correctly. The average age of our cohort was 22.7 years ± 24.8 years SD. The distribution of sex and age when the CT scan was obtained are shown in Table 1.

The ratio of female to male patients in this study was almost 1:1. The youngest participant in this study was 12 months old. A statistical analysis was carried out using a free-to-download statistical function package (R version 3.6.3, IDE: R Studio, Platform: Windows 10) and the results are shown in Table 2.

**Table 2:** Statistical analysis of the measured angles on both sides

	Minimum angle	1st quartile	Median	Mean	3rd quartile	Max. Angle	Standard deviation
Left side	20.5°	34.8°	44.0°	45.0°	53.7°	72.4°	12,0°
Right side	20.9°	40.3°	45.6°	47.2°	53.0°	75.3°	10.4°

**Fig. 4:**
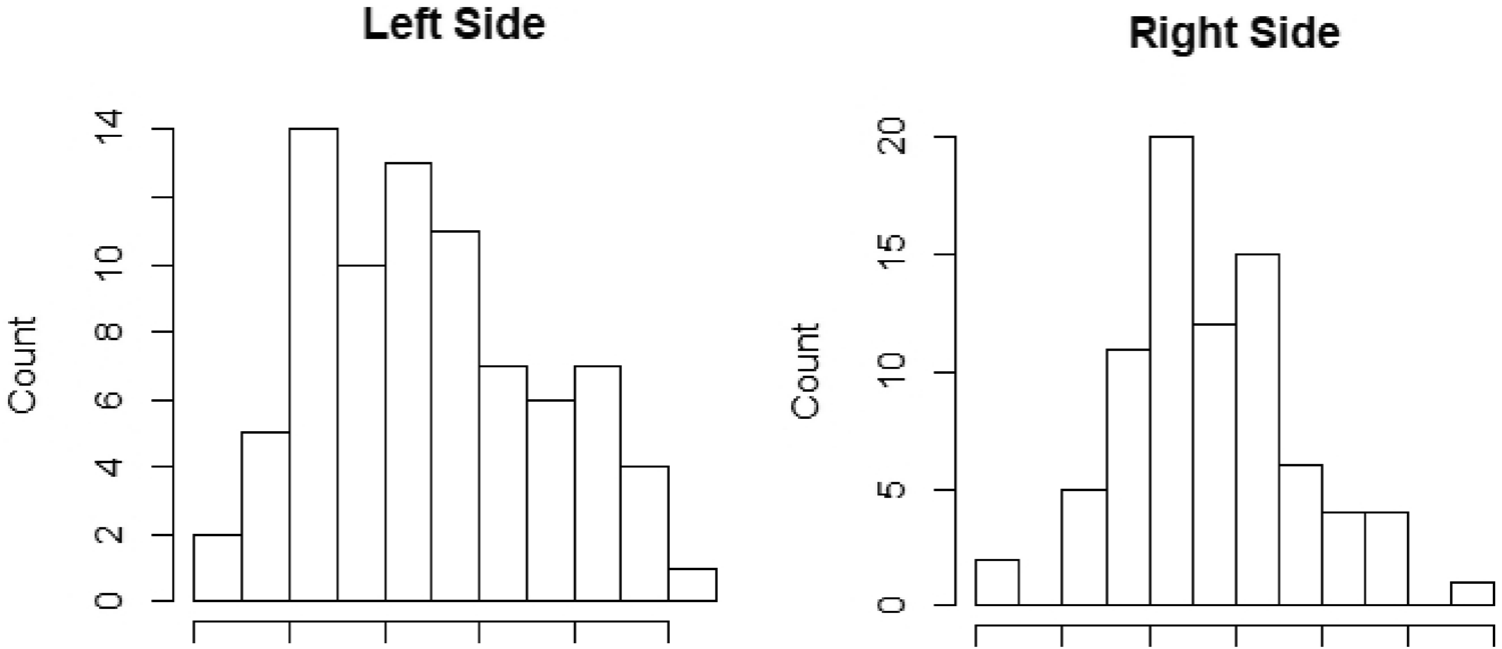
Histogram of the angle distribution on both sides, mean is 45.0 (left side), 47.2 (right side), standard deviation: 12.0 (left side), 10.4 (right side). We established with Shapiro–Wilk test that the data follow normal distribution, *α* = 0.05, *p* values: 0.187 (left side), 0.133 (right side)

There was no significant difference between whether the implantation was carried out on the right or the left side. The average angle was close: 45.0–47.2° ± 10.4–12.0° SD (Table 2 and Fig. 4). Afterwards, a 98% confidence interval was calculated for the mean on both sides. On both sides the *p* value was less than 0.02, thus we can establish that, with 98% probability, the sample mean can represent the population mean. Thus, the expected value of the insertion angle is approximately 45.0–47.2° ± 10.4–12.0° SD.

### Correlation of electrode array insertion angle with the side (left or right) of insertion

**Fig. 5:**
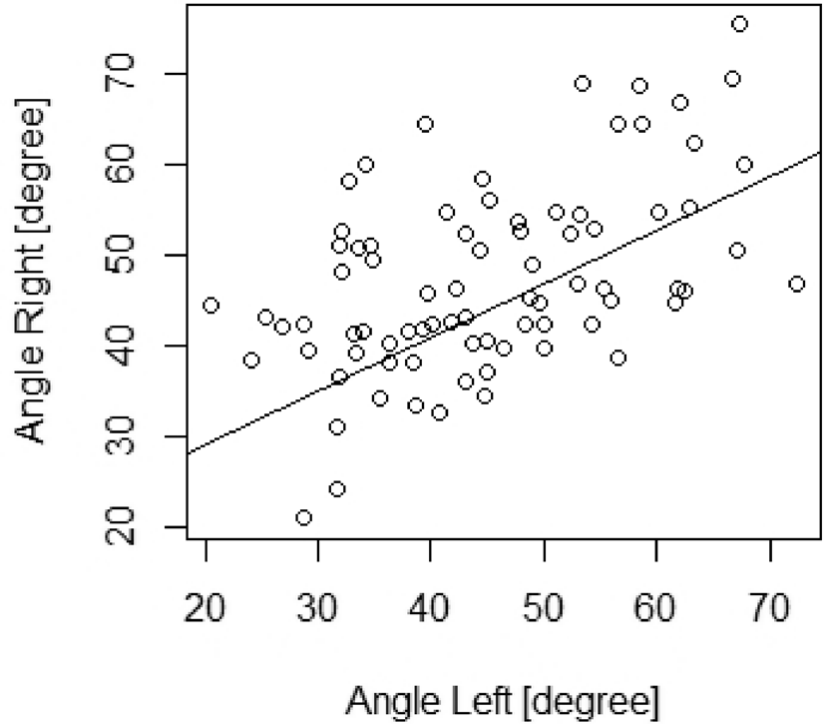
Scatter plot of the angles, showing a weak positive linear connection between the left and right side measurements (Pearson’s correlation coefficient: 0.513) Significance test of the correlation coefficient: coefficient. The Student’s *t*-distribution’s *t* value was 5.271 and *t*_78, 0.05_ = 1.99 (*df* = 78, *α* = 0.05, *p* = 1.172*e *− 06). Because |*t*|> *t*_78, 0.05_ and *p* < *α* the correlation coefficient is significantly different from zero

First, we examined whether there are any linear connections between the left side and right side measurements. For this a Pearson’s correlation test was used. It was confirmed that the values of the angles follow a normal distribution, because this is essential when using Pearson’s correlation test. For this, we used the Shapiro–Wilk test (*α *= 0.05). On the left side, the *p* value was 0.187, whereas on the right side, the *p *value was 0.133 (Fig. 4). Because the *p *values were higher than 0.05, it was accepted that the angles follow normal distribution. The Pearson’s correlation coefficient was 0.513. A significance test was performed for this correlation coefficient. The Student’s *t* distribution value was 5.271 and *t*_78, 0.05_ = 1.99 (*df* = 78, *α* = 0.05, *p* = 1.172*e *− 06). Because |*t*|> *t*_78, 0.05_ and *p* < *α* the correlation coefficient is significantly different from zero, there is a weak positive linear correlation between the measured side and the size of the angle different from zero, which means there is a weak positive linear correlation shown in Fig. 5.

### Correlation of electrode array insertion angle with the sex and age of the patients

Previous studies have raised the possibility of the anatomical differences of the cochlea between females and males [13]. We extended our study to compare the different sexes and the size of the electrode array insertion angle. Before a two-sample *t* test the equality of the variance was assessed. The *p* value of the variance test was 0.135 on the left side and 0.084 on the right side (*α* = 0.01). The equality of variance was accepted due to the *p* > *α* on both sides. Then a two-sample *t* test was performed which included the angle and the sex of the patient (*α* = 0.01). The *p *value on the left side was 0.124 and on the right side it was 0.115. The *p* values were higher than 0.01, therefore the sex of the patient had no statistically significant effect on the size of the angle.

Then it was examined whether the age of the patient has any effect on the calculated angles. Since age is a discrete variable, a one-way Anova test was used. The *p* value on the left side was 0.712 and on the right side it was 0.160. Because the *p* values were higher than 0.05, indicating that age has no statistically significant effect on the size of the angle.

### Electrode array insertion angle in known tip fold-over patients

To compare the electrode array insertion angle in our eighty patients and tip fold-over cases, we determined the insertion angle in five previously implanted patients with confirmed electrode array tip fold-over after the cochlear implantation (Table 3). For this study, preoperative CT scans were used. We then compared the insertion angle where the tip fold-over occurred to the average angle. Although angles of Patient 2 and Patient 3 are very close to the mean, the others (Patient 1, Patient 4 and Patient 5) are close to the endpoint of SD range.

**Table 3:** Tip fold-over cases

	Left side angle [°]	Right side angle [°]
Patient 1	35.4	**34.2**
Patient 2	**44.9**	37.1
Patient 3	52.9	**46.9**
Patient 4	**54.3**	42.2
Patient 5	**55.9**	44.9

The angles determined in the five patients where tip fold-over occurred. These patients had bilateral cochlear implantations and the angles in bold are the side where the tip fold-over occurred

## Discussion

This study investigated the average OM orientation to anatomical landmarks, determined by a custom-made Python script, in eighty cochlear implanted patients’ preoperative CT scans. The statistical analysis indicated that the alignment of the electrode array in a successful CI insertion is approximately 45.0° ± 11° SD. Furthermore, there is no impact of the age or the sex of the patients on the insertion angle. However, there was a weak positive linear correlation observed between the left and right sides.

### Calculation of insertion angle

3D Slicer software is able to mark anatomical structures, such as the short process of the incus (ISP) on the axial plane of a CT scan. When changed to the coronal plane, it could be rotated to the cochlear view, where the insertion guide (IR) and the orientation marker (OM) could be marked and measured. These 3D points and vectors were loaded into a custom python scripted 3D Slicer module, which was used to calculate the angle. The limitation of this technique is the manual measurements on the CT scans. If the user cannot mark the landmarks or create the exact cochlear view, the angles may be distorted.

With the advancement of imaging techniques and software (e.g. 3D Slicer, RadiAnt DICOM viewer), it is relatively easy to identify anatomical landmarks on a CT scan and calculate the insertion angle. It was previously demonstrated that an anatomical landmark-based approach such as using the centre of the round window at the bony overhang, the basal and apical centre of the modiolus, can be used as a cochleostomy target [14]. This consequently provided valuable information for an image-guided robotic system to carry out the exact surgical drilling based on the estimated optimal trajectory. In this study, the ideal insertion angle of the CI electrode array into the cochlea was identified. However, it would be useful to incorporate the positioning of the cochleostomy that would guide the implant electrode array into the cochlea.

### Insertion angle in tip fold-over cases

Until recently, tip fold-over of the electrode array was only small probability (~ 0,80%) seen in lateral wall electrode arrays. However, with the new thin perimodiolar electrode array model, tip fold-over can occur in ~ 4.7% of cochlear implanted cases [8].

In this study, we calculated that the average angle of the OM to the ISP in successful implantations is approximately 45.0–47.2° ± 10.4–12° SD which was verified with a confidence interval of 98%. Furthermore, after determining the insertion angle on five known tip fold-over cases’ preoperative CT scans, the angles did fall within the average range. Although the values suggested that the further the diversion from 45.0° to 47.0° ± SD, the increased chance of tip fold-over. However, in Patient 2 and Patient 3 the measured angles were in the average range, it is possible that the tip fold-over was caused by factors other than the insertion angle alone.

Based on our experience, other contributing factors are likely to include: (i) too fast or forced insertion of the very delicate electrode array [15], (ii) incomplete loading of the array with the tip remaining and curving already outside the IG, (iii) incorrect loading of the electrode array which causes the array to stuck in the slot of the IG, (iv) incorrect insertion trajectory vector, for example, if the array is directed too much towards the medial or lateral wall of the cochlea which also may cause bending of the IG. In this situation, the deformed IG’s slot may expand which results in electrode array insertional failure. It is assumed that incorrect insertion trajectory can also be caused by a narrow or insufficiently extended RW or the presence of a pronounced fissula ante-fenestram.

### Potential impact and future applications

Anatomical landmark identification during electrode array insertion in CI surgery has been continuously investigated. A study by Meshnik et al. [16] used eight cadaveric human temporal bones and applied a similar technique to our study: the fusion of microCT imaging with Analyze imaging software analysis alongside a custom-written script to determine five possible insertion vectors for the most optimal electrode array site of insertion. However, their study was tailored for cochleostomy approach, therefore, it is not possible to make a direct comparison. The close relationship of the optimal insertion vectors to the facial nerve warrants an investigation to assess whether the optimal angle of orientation identified in this study may also need to take into consideration the location of the facial nerve.

Furthermore, anatomical landmark guidance is becoming important for the future of CI surgery due to technological advancements and improvements in surgical techniques. The CI632 implant user guide [17] lacks a precise numerical recommendation on where the orientation marker should point during the insertion of the electrode array, which may lead to potential misalignment. Our study intended to quantify the optimal position of the orientation marker relative to the visible intraoperative anatomical landmarks. Furthermore, a numerical approach is likely to aid in personalising the electrode array insertion, since the basal turn of the cochlea may vary between individuals. This may also reduce the chance of damaging the basilar membrane by the electrode array, which could result in decreased residual hearing preservation [18]. The use of landmark based and numerical approach is also very valuable for training less experienced surgeons to standardise the process leading to improved consistency through an evidence-based approach [19].

Preserving residual hearing and developing a less traumatic insertion of the electrode array have been main goals for many institutions, which could potentially be achieved using robotic surgery [20–22]. There is limited knowledge on post-robotic insertion hearing outcomes, but recent studies have shown that the robot itself is able to decrease the involuntary movements such as tremor; creates a smooth insertion and the translocated electrodes were decreased in comparison to manual insertion with reduced intracochlear damage, however, navigation and preoperative planning are still under refinement. The optimal angle of orientation identified in the current study, together with the anatomical reference points for electrode array with proven consistent clinical outcomes, could aid the development of preoperative input data for personalised robotic array insertion.

## Conclusion

Due to the differences in the individual anatomy, this ~ 45.0°–47.0° ± SD angle range should not be applied automatically for all cases. Although there is a weak positive correlation between the values of left and right side angles, it is necessary to take measurements bilaterally if both sides are implanted. Although this method was developed for the Slim Modiolar electrode, this method could be adopted to other electrode arrays with half-band electrodes (e.g. the Cochlear™ Nucleus^®^ Slim Straight and Contour Advance). If half-band electrodes are used, the position of the OM related to the position of the modiolus should be considered and the calculated angle should be corrected accordingly (e.g. 180° should be added if the marker or guidewire is to be positioned caudally). The full-band electrode types, however, do not require such measurements, because their design allows their insertion in any orientation angle (e.g. MEDEL^®^: FORM^®^ and CLASSIC^®^ Series). Our results can serve as valuable additional information for the surgeon in planning and performing the implantation procedure. During electrode array insertion, the plane of the basal turn of the cochlea is not visible. The 3D models and the calculated angles provide deeper knowledge of the individual anatomy pre-operatively. Before the insertion of the electrode array into the RW, the surgeon can align the OM towards the ISP using the patients’ preoperative calculated angle. This angle considers the individual anatomy of the patient and guides the surgeon based on visible anatomical landmarks. Thus, using the quantified angle, the surgeon does not have to rely exclusively on intuition of the cochlear basal turn during orientation. Furthermore, consideration of cochlear anatomy during electrode array orientation potentially reduces complications such as tip fold-over and interscalar dislocation.

## Data Availability

The datasets used and/or analysed during the current study are available from the corresponding author on reasonable request.

## Abbreviations

CI: Cochlear implant
CT: Computed Tomography
IG: Insertion Guide
ISP: Incus short process
OM: Orientation marker
RW: Round window
SD: Standard deviation
